# Excessive cholecalciferol supplementation increases kidney dysfunction associated with intrarenal artery calcification in obese insulin-resistant mice

**DOI:** 10.1038/s41598-019-55501-3

**Published:** 2020-01-09

**Authors:** Youri E. Almeida, Melissa R. Fessel, Luciana Simão do Carmo, Vanda Jorgetti, Elisângela Farias-Silva, Luciana Alves Pescatore, Lionel F. Gamarra, Maria Claudina Andrade, Antonio Simplicio-Filho, Cristóvão Luis Pitangueiras Mangueira, Érika B. Rangel, Marcel Liberman

**Affiliations:** 1 Hospital Israelita Albert Einstein, São Paulo/SP, 01425001 Brazil; 2 Laboratório de Biologia Vascular, LIM-64, InCor, Hospital das Clinicas HCFMUSP, Faculdade de Medicina, Universidade de Sao Paulo, São Paulo/SP, Brazil; 3 Department of Nephrology, Medical School, Universidade de São Paulo, São Paulo/SP, 01246000 Brazil

**Keywords:** Calcification, Diabetes complications, Type 2 diabetes, Obesity, End-stage renal disease

## Abstract

Diabetes mellitus accelerates vascular calcification (VC) and increases the risk of end-stage renal disease (ESRD). Nevertheless, the impact of VC in renal disease progression in type 2 diabetes mellitus (T2DM) is poorly understood. We addressed the effect of VC and mechanisms involved in renal dysfunction in a murine model of insulin resistance and obesity *(ob/ob)*, comparing with their healthy littermates (C57BL/6). We analyzed VC and renal function in both mouse strains after challenging them with Vitamin D_3_ (VitD_3_). Although VitD_3_ similarly increased serum calcium and induced bone disease in both strains, 24-hour urine volume and creatinine pronouncedly decreased only in *ob/ob* mice. Moreover, *ob/ob* increased urinary albumin/creatinine ratio (ACR), indicating kidney dysfunction. In parallel, *ob/ob* developed extensive intrarenal VC after VitD_3_. Coincidently with increased intrarenal vascular mineralization, our results demonstrated that Bone Morphogenetic Protein-2 (BMP-2) was highly expressed in these arteries exclusively in *ob/ob*. These data depict a greater susceptibility of *ob/ob* mice to develop renal disease after VitD_3_ in comparison to paired C57BL/6. In conclusion, this study unfolds novel mechanisms of progressive renal dysfunction in diabetes mellitus (DM) after VitD_3_ *in vivo* associated with increased intrarenal VC and highlights possible harmful effects of long-term supplementation of VitD_3_ in this population.

## Introduction

Vascular calcification (VC) is a pathological condition which causes loss of arterial elasticity and augments vascular stiffening associated with increased cardiac work^1,2^. This contributes to several cardiovascular diseases such as systemic arterial hypertension, coronary artery and cerebrovascular disease, congestive heart failure, and end-stage renal disease (ESRD)^3^. Previously thought as a process of physiological aging, VC is currently known as an active and complex process, characterized by increased calcifying signaling agonists that overcome inhibitory mediators and resembles skeleton ossification^1,2^.

Diabetes mellitus is a huge public health problem in the world. It is estimated that diabetic population will reach 430 million people in 2030. Incidence of ESRD is up to 10 times as high in adults with diabetes as those without^4^. ESRD attributable to diabetes is 12–55%^4^. In the last decades, numerous advances have been incorporated to the treatment of ESRD. However, the incidence of renal replacement therapy and mortality rates in this population remains high^5,6^.

Epidemiological studies describe ESRD and VC as independent cardiovascular risk factors^5^. However, ESRD patients are usually identified with VC^7,8^, and ESRD together with diabetes mellitus accelerates VC progression^7–9^. Studies that examined VC in the kidney are scarce and the impact of VC influencing renal disease progression in diabetes mellitus is poorly understood.

Active 1,25-dihydroxyvitamin D_3_ is implicated in prevention and treatment of hyperparathyroidism in dialysis patients. In addition, there is accumulating evidence that VitD_3_ has a beneficial role in decreasing proteinuria^10^, the hallmark of diabetic nephropathy and other chronic kidney diseases. VitD_3_ may primarily suppress renin-angiotensin-aldosterone system, reduce the inflammatory response, apoptosis, fibrosis, and oxidative stress, and mitigate podocyte cytoskeleton damage^10,11^. Furthermore, nonskeletal benefits of Vitamin D supplementation was motivated by reports associating low levels of VitD_3_ and development of pathological conditions such as metabolic syndrome, ESRD, and type 2 diabetes mellitus^12^. Paradoxically, inappropriate doses of vitamin D, mainly associated with clinical malpractice, can trigger acute kidney injury^13^, which ultimately lead to permanent kidney damage due to fibrosis, and exaggerated VC (aorta) in a model of monogenic obesity and insulin resistance^14,15^. To the best of our knowledge, the renal function of individuals subjected to high doses of VitD_3_ was not previously assessed. Furthermore, no large clinical trials were conducted to investigate harmful effects of Vitamin D supplementation, especially in diabetes mellitus, ESRD and elderly patients. From this perspective, the aim of this study was to address the effect of VitD_3_-induced intrarenal VC and respective mechanisms involved in renal dysfunction in a murine model of insulin resistance and obesity (*ob/ob*), comparing to paired healthy littermates (C57BL/6). Accordingly, based on a previous study that demonstrated increased aortic calcification in *ob/ob* mice after excessive VitD_3_ supplementation^14^, we examined the contribution of renal arteries calcification in CKD progression both in *ob/ob* and in C57BL/6 mice.

## Results

### Diabetic *ob/ob* mice developed Vitamin D_3_-induced kidney dysfunction

**Figure 1:**
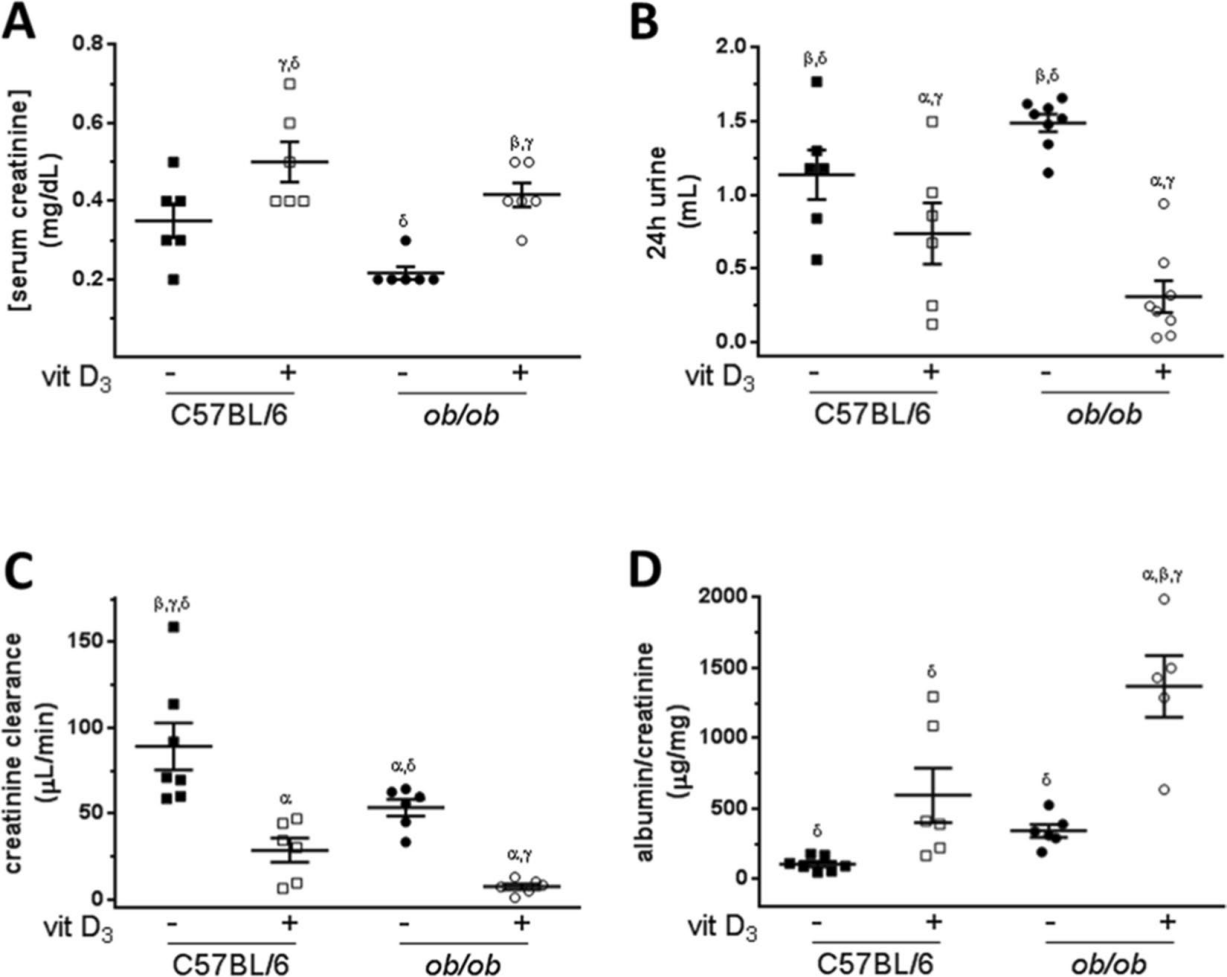
Renal function analysis of obese insulin-resistant mice (*ob/ob*) and their littermates (C57BL/6) subjected to Vitamin D_3_ protocol. (**A**) Serum creatinine concentration (n = 5–6); (**B**) 24-hour urine volume quantification (n = 6–8); (**C**) Creatinine clearance levels evaluation (n = 5–7) and (**D**) albumin/creatinine ratio determination (n = 5–7) from mice treated (+) with Vitamin D_3_ or injected with saline (−). α, β, γ, δ = *P* < 0.05, in comparison to untreated (−) C57BL/6, treated (+) C57BL/6, untreated (−) *ob/ob*, and treated (+) *ob/ob* respectively.

Both saline-treated C57BL/6 and *ob/ob* mice presented similar serum creatinine levels (0.35 ± 0.10 mg/dL *versus* 0.22 ± 0.04 mg/dL, respectively), Fig. 1A. After VitD_3_ protocol, serum creatinine increased 92% (0.42 ± 0.03 mg/dL) and 43% (0.50 ± 0.05 mg/dL) in *ob/ob* and C57BL/6 mice respectively (Fig. 1A) *versus* paired controls. Enhanced creatinine levels in both strains indicate kidney dysfunction induced by hypervitaminosis D. In parallel, saline-treated *ob/ob* mice showed lower creatinine clearance than saline-injected C57BL/6 mice (53.5 ± 4.9 µL/min *vs*. 89.1 ± 13.7 µL/min), Fig. 1C. After VitD_3_ protocol, creatinine clearance decreased in both strains, more pronouncedly in *ob/ob* (7.61 ± 1.69 µL/min) in comparison to C57BL/6 mice (28.74 ± 7.01 µL/min), Fig. 1C.

To further investigate the effects of VitD_3_ protocol in renal dysfunction, we assessed 24h-urine volume output, albuminuria and calculated ACR to estimate glomerular damage, which also depicts progressive renal disease in diabetes mellitus^16^. As expected, the diabetic model showed increased 24h-urine volume in comparison to C57BL/6 (1.49 ± 0.06 mL *vs*. 1.14 ± 0.17 mL) at baseline. After 21 days of VitD_3_ treatment, both *ob/ob* and C57BL/6 strains presented decreased 24h-urine volume. This was more pronounced in *ob/ob* (0.31 ± 0.11 mL) than in C57BL/6 mice (0.74 ± 0.21 mL), Fig. 1B.

Albumin creatinine ratio was similar both in saline-treated C57BL/6 and in *ob/ob* mice (106.9 ± 19.1 µg/mg and 340.9 ± 45.4 µg/mg, respectively). ACR from VitD_3_-treated and from saline-treated C57BL/6 mice were not statistically different (585.1 ± 194.7 µg/mg and 106.9 ± 19.1 µg/mg, respectively), Fig. 1D. Interestingly, ACR from *ob/ob* VitD_3_-treated mice increased in comparison to paired saline-treated *ob/ob* mice (1369.9 ± 218.0 µg/mg *vs*. 340.9 ± 45.4 µg/mg) and to C57BL/6 VitD_3_-treated mice (585.1 ± 194.7 µg/mg), Fig. 1D.

### High dose of Vitamin D_3_ induced similar bone disease in both *ob/ob* and C57BL/6 mice

VitD_3_ treatment increased ≈70% trabecular volume (BV/TV) in both *ob/ob* and C57BL/6, Table 1. Moreover, both strains augmented trabecular thickness (Tb.Th); this response was 20% less in *ob/ob* compared to C57BL/6. Both trabecular number (Tb.N) and trabecular separation (Tb.Sp) did not significantly change after VitD_3_ administration in C57BL/6 and *ob/ob* mice, Table 1. Static parameters of bone formation showed that VitD_3_-treated mice increased osteoid surface (OS/BS) in both strains (9 times and 8 times increase in C57BL/6 and in *ob/ob*, respectively). Similarly, VitD_3_ enhanced osteoid volume (OV/BV) in both strains (≈50 times for C57BL/6 and ≈35 times for *ob/ob*), Table 1. Femurs also presented osteoid thickness modification (O.Th) after hypervitaminosis D. Specifically, C57BL/6 and diabetic *ob/ob* mice increased (25% and 50%, respectively) osteoid thickness in comparison to paired controls. Osteoblastic surface (Ob.S/BS) enhanced after VitD_3_ in both strains, especially in *ob/ob* mice (≈6 times and. ≈3 times in *ob/ob* and C57BL/6 mice respectively), Table 1. Finally, bone resorption parameters demonstrated that osteoclast surface (Oc.S/BS) from *ob/ob* control femurs was approximately 50% inferior in comparison to C57BL/6 controls. VitD_3_ reduced Oc.S/BS value (C57BL/6 = 27% and *ob/ob* = 16%). Eroded surface (ES/BS) from control *ob/ob* mice was 40% less than in saline-injected C57BL/6 animals. ES/BS decreased after VitD_3_ in both strains (C57BL/6 = 19% and *ob/ob* = 14%), Table 1.

**Table 1:** Femur’s Histomorphometric parameters from C57BL/6 and from *ob/ob* mice subjected to Vitamin D_3_ (+) or to saline (−).

Parameter	C57BL/6		*ob/ob*	
	−	+	−	+
BV/TV (%)	14.95 ± 1.27	25.80 ± 0.93^a^	10.36 ± 1.53^b^	17.81 ± 1.84^b^
Tb.Th (µm)	31.42 ± 1.46	57.37 ± 2.34^a^	31.03 ± 0.20^b^	45.61 ± 2.93^a,b,c^
Tb.N (n°/mm)	4.71 ± 0.19	4.53 ± 0.24	3.33 ± 0.47	3.86 ± 0.26
Tb.Sp (µm)	182.4 ± 10.2	166.3 ± 10.1	274.9 ± 43.6^b^	220.6 ± 21.7
OS/BS (%)	9.13 ± 1.66	81.63 ± 3.77^a^	10.59 ± 2.52^b^	78.47 ± 5.00^a,c^
OV/BV (%)	0.82 ± 0.18	41.54 ± 0.86^a^	1.22 ± 0.62^b^	42.32 ± 2.87^a,c^
O.Th (µm)	1.37 ± 0.12	15.66 ± 0.76^a^	1.74 ± 0.50^b^	13.10 ± 0.90^a,c^
Ob.S/BS (%)	7.38 ± 1.25	22.77 ± 2.09^a^	4.84 ± 2.28^b^	29.56 ± 4.38^a,c^
Oc.S/BS (%)	1.72 ± 0.14	0.46 ± 0.19^a^	0.79 ± 0.52^a^	0.12 ± 0.05^a^
ES/BS (%)	7.21 ± 0.62	1.40 ± 0.39^a^	2.84 ± 0.66^a^	0.40 ± 0.16^a,c^

^a^*P* < 0.05 in comparison to C57BL/6 control.

^b^*P* < 0.05 in comparison to C57BL/6 Vitamin D_3_-treated.

^c^*P* < 0.05 in comparison to *ob/ob* control.

High dose of Vitamin D_3_ induced similar damage of bone tissue in both *ob/ob* and in C57BL/6 mice, as demonstrated by static bone formation parameters and bone resorption data from these animals *n* = 3–6 (animals injected with saline (−)); *n* = 6–7 (for animals treated with VitD_3_ (+)). Increased osteoid matrix and resorption parameters characterize a mixed bone disease.

### Vitamin D_3_ induced intrarenal artery calcification in diabetic mice, but not in paired C57BL/6 littermates

**Figure 2:**
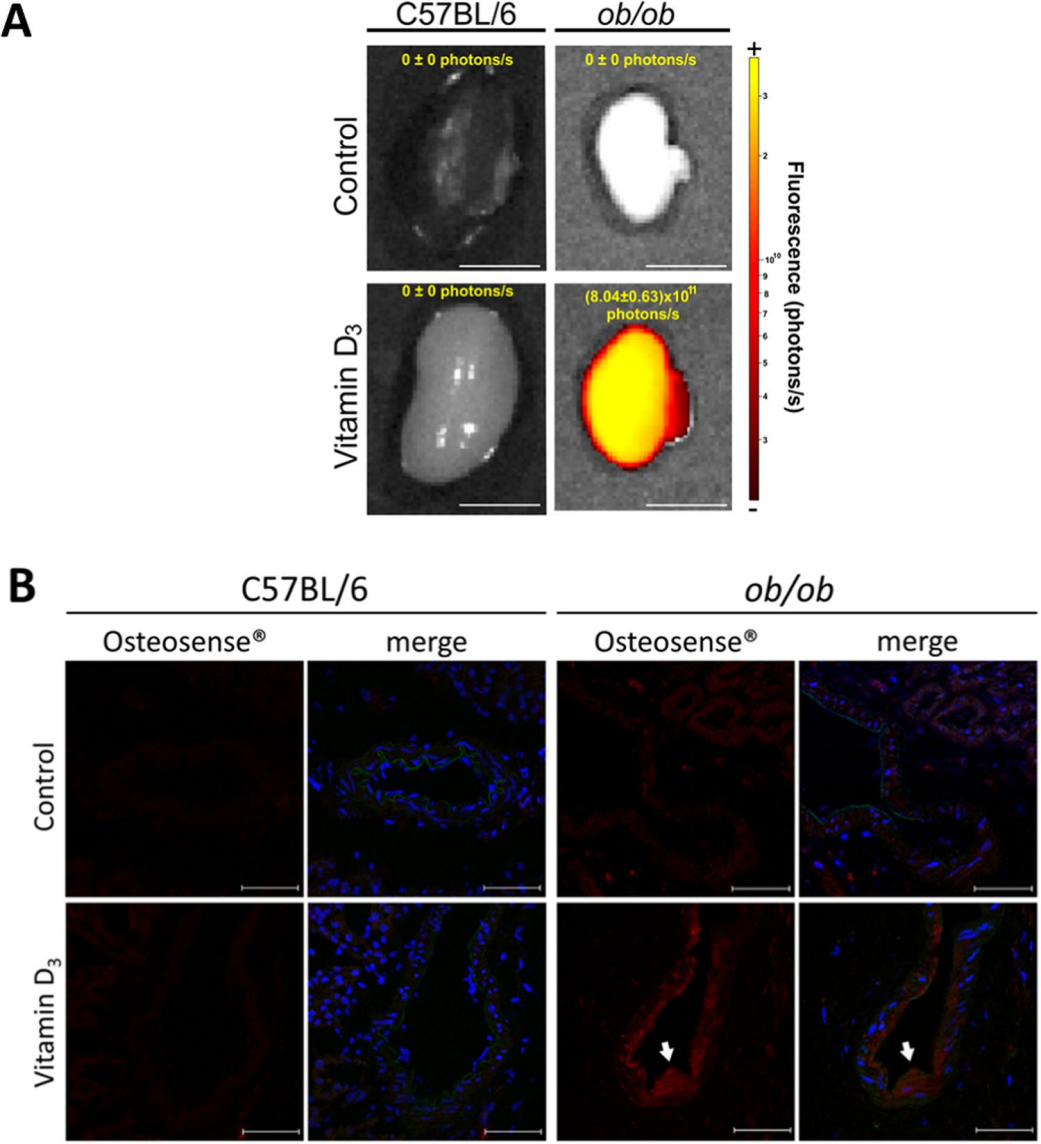
Vitamin D_3_ increased calcification in obese insulin-resistant (*ob/ob*) mice but not in their littermates (C57BL/6) - Mice were subjected to Vitamin D_3_ treatment (Vitamin D_3_) or saline administration (control). 24 h before euthanasia, Osteosense 680 EX was injected i.p., kidneys were isolated and analyzed. (**A**) Kidneys were imaged *ex vivo* as described in methods section. Right: fluorescence intensity scale, photon/s. Only kidneys from Vitamin D_3_-treated *ob/ob* mice exhibited high fluorescence (average mean and standard error values are indicated as a picture insert), n = 3. Bars = 5 mm. (**B**) Histological slides of the kidneys were analyzed in a confocal fluorescence microscope. Osteosense 680 EX is depicted in red and nuclei Hoechst-stained are shown in blue. Arrows depict high Osteosense 680 EX signal staining calcification of medial layer of intrarenal arteries from *ob/ob* mice. Mean Fluorescence intensity normalized by C57BL/6 control: 1.0; 1.2; 1.1 and 2.8 in C57BL/6 control; *ob/ob* control; C57BL/6 Vitamin D_3_ and *ob/ob* Vitamin D_3_ respectively, n = 2. Bars = 50 µm.

*Ex-vivo* kidney analysis exhibited high intensity Osteosense-derived fluorescence in VitD_3_-treated *ob/ob* mice, but not in VitD_3_-treated-C57BL/6 nor in paired saline-injected mice, thus demonstrating greater calcification (Fig. 2A). In addition, confocal fluorescence microscopy coincidently showed high-intensity Osteosense 680 EX signal in medial layer of intrarenal arteries from *ob/ob* VitD_3_-treated mice, but not in paired VitD_3_-treated C57BL/6 mice (Fig. 2B).

**Figure 3:**
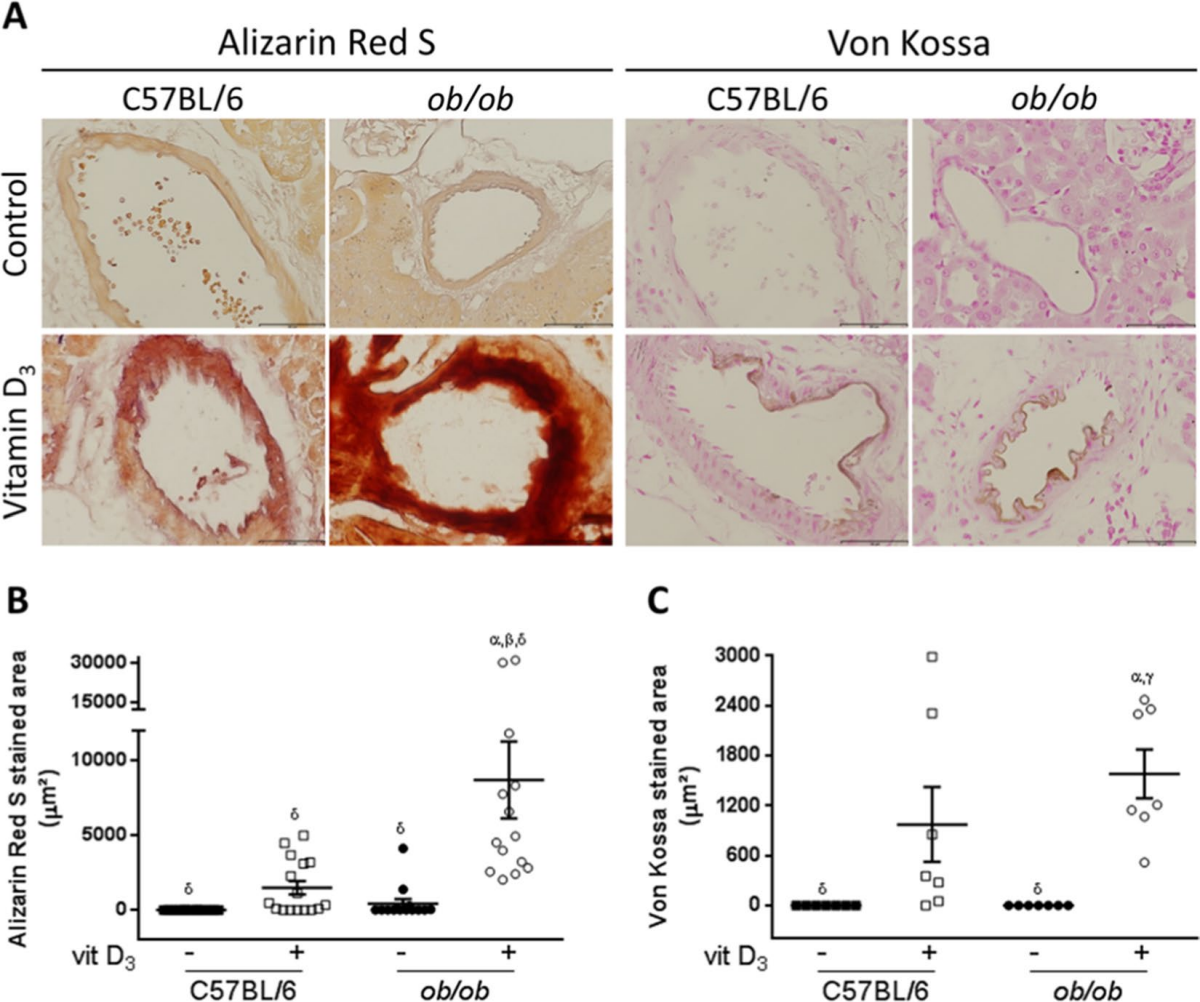
Histochemical analysis and quantification of intrarenal arteries calcification from obese insulin-resistant *ob/ob* mice and their littermates C57BL/6 subjected to Vitamin D_3_ protocol. (**A**) Mice were subjected to Vitamin D_3_ treatment (Vitamin D_3_) or saline administration (Control) and tissue secctions were stained with Alizarin Red S and Von Kossa. Both Alizarin Red S (left panels) and Von Kossa (right panels) staining demonstrated increased calcification of intrarenal arteries from VitD_3_-treated *ob/ob* in comparison to paired C57BL/6 mice. (**B**,**C**) Quantification of stained areas for Alizarin Red S (**B**) and Von Kossa (**C**) staining, in µm^2^, from mice treated with Vitamin D_3_ (+) or injected with saline (−). α, β, γ, δ = *P* < 0.05, in comparison to saline (−) C57BL/6, VitD_3_-treated (+) C57BL/6, saline (−) *ob/ob*, and VitD_3_-treated (+) *ob/ob*, respectively. *n* = 6 for all groups.

We further confirmed these findings using histochemical techniques. Diabetic *ob/ob* mice showed increased Alizarin Red S and Von Kossa staining in intrarenal arteries after VitD_3_ treatment compared to the paired C57BL/6 (Fig. 3A). Negligible or no calcification was identified in intrarenal arteries from saline-treated *ob/ob* and C57BL/6 mice (Fig. 3A). Quantification of Alizarin Red S staining confirmed these findings: 8717 ± 1.71 µm^2^ *vs*. 416 ± 1.71 µm^2^ in VitD_3_-treated *vs*. saline-treated *ob/ob* mice; 1479 ± 1.66 µm^2^ *vs*. 0 ± 1.66 µm^2^ in VitD_3_-treated *vs*. saline-treated C57BL/6 respectively (Fig. 3B). Accordingly, quantification of Von Kossa staining showed: 1582 ± 380.7 µm^2^ *vs*. 0 ± 380.7 µm^2^ in VitD_3_-treated *ob/ob* and saline-treated *ob/ob* mice; 975.9 ± 380.7 µm^2^ *vs*. 0.0 ± 380.7 µm^2^ in VitD_3_-treated C57BL/6 and saline-treated C57BL/6 mice respectively (Fig. 3C). In conclusion, these data showed greater calcification response of intrarenal arteries from *ob/ob* than from paired C57BL/6 after VitD_3_ protocol.

### BMP-2 is highly expressed on calcified intrarenal artery from Vitamin D_3_-treated diabetic mice

**Figure 4:**
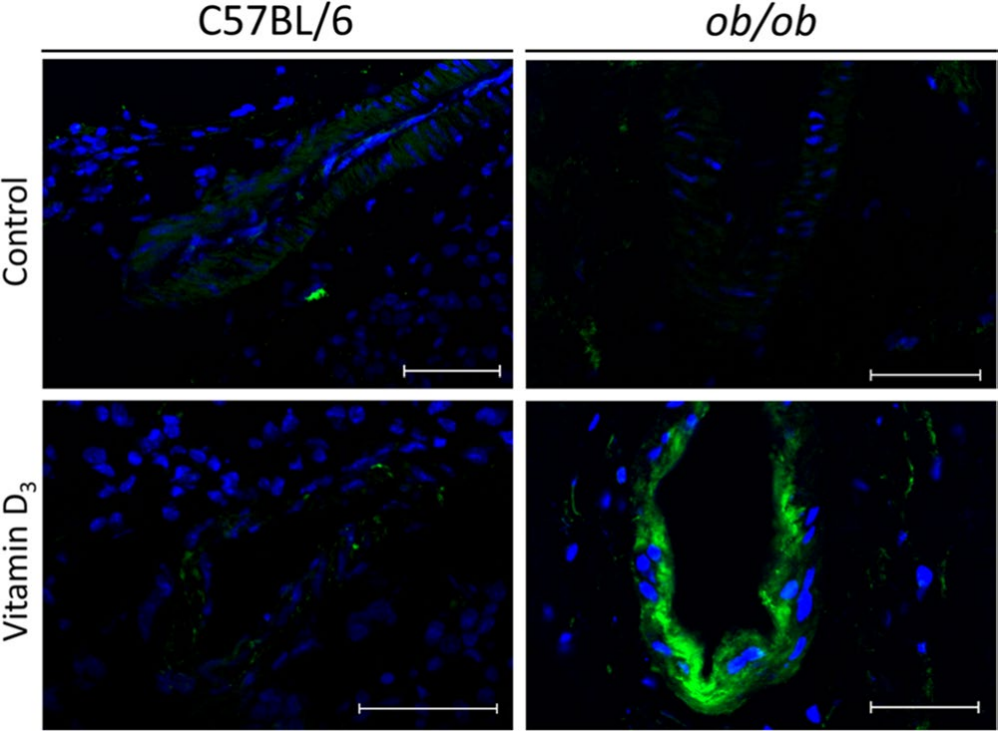
Immunofluorescence analysis of BMP-2 expression in intrarenal arteries from obese insulin-resistant *ob/ob* mice and their littermates C57BL/6 subjected to Vitamin D_3_ protocol. Mice were subjected to Vitamin D_3_ treatment (Vitamin D_3_) or injected with saline (control). Kidney sections were incubated with anti-BMP-2, followed by secondary AlexaFluor 488-conjugated antibody incubation and analyzed by confocal fluorescence microscopy. Increased BMP-2 expression (green) is observed in calcified intrarenal arteries from Vitamin D_3_–treated *ob/ob* mice. On contrary, paired C57BL/6 mice exhibited low BMP-2 expression. Nuclei were stained with Hoechst 33342 (blue). Mean fluorescence intensity and standard error of samples are: 5.32 ± 5.32; 65.95 ± 58.36; 87.08 ± 40.03 and 235.80 ± 21.09 in C57BL/6 control; C57BL/6 Vitamin D_3;_ *ob/ob* control and *ob/ob* Vitamin D_3_ respectively. P < 0.05, *ob/ob* Vitamin D_3_ *versus* all groups, n = 3–4. Bars = 50 µm.

As we consistently demonstrated increased VC induced by VitD_3_ in diabetic mice (Figs. 2 and 3), we evaluated BMP-2 expression, which has a pivotal role in smooth muscle cells osteochondrogenic dedifferentiation and ectopic calcification^17,18^. We found that BMP-2 was highly expressed in intrarenal arteries exclusively from *ob/ob* mice subjected to VitD_3_ protocol (Fig. 4), but not in paired VitD_3_-injected C57BL/6 samples.

### Severe mesangial expansion associated with acute tubular necrosis in VitD_3_-treated *ob/ob* mice

**Figure 5:**
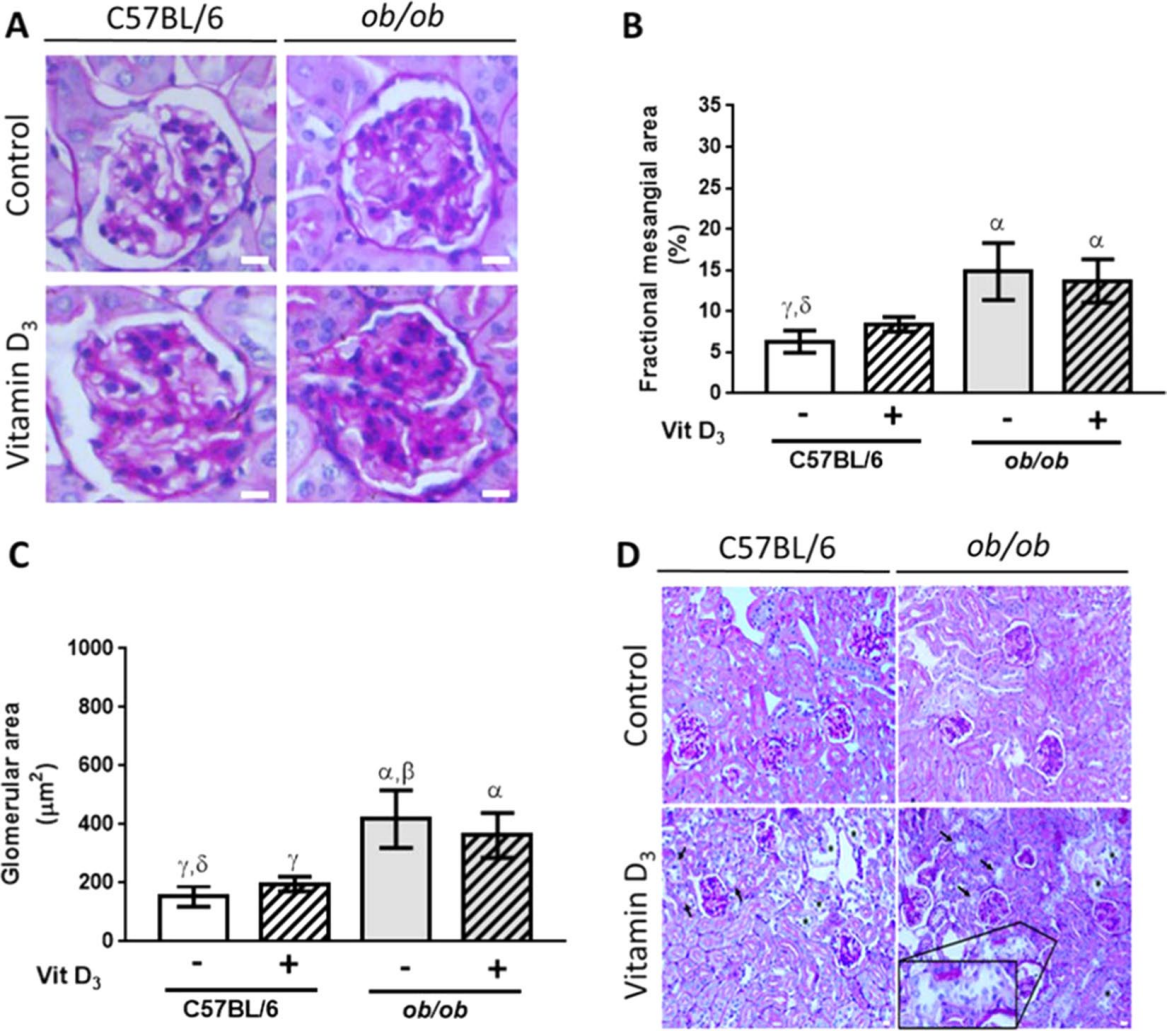
Mesangial and histological assessment of the kidneys showing severe mesangial expansion in *ob/ob* mice, and the effect of Vitamin D_3_ in glomerular damage and in acute tubular necrosis. (**A**) PAS staining of glomeruli from C57BL/6 and from *ob/ob* mice after Vitamin D_3_ stimulation (+) or after saline injection (−). Bars = 20 µm. (**B**) VitD_3_ promoted increased matrix deposition in mesangial compartment of C57BL/6 mice that was able to reach ob/ob levels (no statistical difference among VitD_3_-treated C57BL/6 and VitD_3_-treated *ob/ob* mice). (**C**) Increased glomerular area in *ob/ob* mice. There was no additive effect of Vitamin D_3_ in ob/ob mice, but VitD_3_ was able to equalize glomerular area in C57BL/6 and in *ob/ob* mice. α, β, γ, δ = *P* < 0.05, in comparison to saline-treated C57BL/6 (n = 9), VitD_3_-treated C57BL/6 (n = 7), saline-treated *ob/ob* (n = 7), and VitD_3_-treated *ob/ob* mice (n = 8), respectively. (**D**) Vitamin D_3_ induced acute tubular necrosis (ATN) both in C57BL/6 and in *ob/ob* mice, as shown by flattening of the renal tubular cells due to tubular dilation (asterisks), loss of brush border (arrows), and degenerative changes characterized by diffuse denudation of the renal cells, presence of necrotic cells, and cellular debris. Insert depicts degenerative changes in a distal tubule. Bars = 20 µm. For saline-injected mice n = 4–6, and for Vitamin D_3_–treated mice, n = 5–8.

In diabetic *ob/ob* mice, mesangial expansion was more severe when compared to wild type mice, Fig. 5A. Of note, VitD_3_ accelerated extracellular matrix deposition in mesangial compartment of C57BL/6, demonstrated by statistically similar values of fractional mesangial area in VitD_3_-treated C57BL/6 compared to VitD_3_-treated *ob/ob* mice, Fig. 5B. Both VitD_3_-treated C57BL/6 and VitD_3_-treated *ob/ob* mice’s fractional mesangial area did not show statistical difference when compared to paired saline-treated C57BL/6 and *ob/ob* mice respectively, Fig. 5B. These findings may suggest a possible effect of inappropriate high-dose of VitD_3_ in regulating glomerular damage in C57BL/6 mice, since VitD_3_ promoted increased matrix deposition in mesangial compartment that was able to reach *ob/ob* levels. However, in pre-existing diabetic nephropathy settings, in which several deleterious pathways have already been activated, VitD_3_ did not promote additional damage to the mesangial compartment. In parallel, we showed increased glomerular area in *ob/ob* mice. There was no additive effect of VitD_3_ in *ob/ob* mice, but VitD_3_ was able to equalize glomerular area in C57BL/6 and in *ob/ob* mice, Fig. 5C.

Importantly, VitD_3_ also induced acute kidney injury, e.g., ATN both in *ob/ob* and in wild type mice. Cortical and corticomedular tubules showed widespread degenerative changes with luminal dilation and loss of brush border, as well as tubular atrophy, Fig. 5D. These findings may explain the observation of higher serum creatinine levels and lower creatinine clearance and diuresis volume in VitD_3_-treated animals. Importantly, increased mesangial area in *ob/ob* mice combined with ATN lesions may explain higher levels of albuminuria, impairing kidney function in these animals.

Collagen I and III deposition in tubule-interstitial area, as demonstrated by Picro Sirius Red staining, was mild in all groups, but slightly increased in VitD_3_-treated *ob/ob* mice, Supplemental Fig. I.

## Discussion

There is no doubt that the association of DM, VC and ESRD increases morbidity and mortality^19^. In order to investigate kidney-specific VC mechanisms that may implicate in diabetes mellitus-related kidney dysfunction and pathophysiology, we studied the effect of high dose VitD_3_ i.p. injection using an experimental model that mimics T2DM and insulin resistance, the *ob/ob* mouse. Recently, we demonstrated that VitD_3_ increased expansive vascular remodeling associated with accelerated VC in *ob/ob* mice, which occurred by the convergence of increased oxidative stress, matrix metalloproteinase activation and absence of VDR downregulation in the vascular wall from these animals^14^. Conversely, VitD_3_ and its analogous have been reported as renoprotective, resulting in an attenuation of proteinuria, inflammation, glomerulosclerosis and interstitial fibrosis and an improvement of glomerular ratio^20,21^. Reciprocal benefits include renin-angiotensin-aldosterone system regulation, anti-inflammatory, anti-oxidative stress and anti-apoptosis properties, podocyte protection, autophagy activation, immunomodulatory effects, hepatocyte growth factor induction, mitochondrial function regulation, and tubular epithelium preservation, via epithelial-mesenchymal transition blockade^20–22^. Vitamin D_3_ prescription has been increasing and its inappropriate supplementation may be associated with toxicity and hypercalcemia^23^. Recently, investigators reported an interesting ESRD murine model^24^, using phosphorus-rich diet, but it requires twice more time (6 weeks) to develop compared to our model, besides not describing intrarenal arteries calcification. In our study we demonstrated that: 1) VitD_3_ additionally impacted in diabetic mouse model’s renal function and early markers of glomerular filtration damage, by decreasing creatinine clearance, 24 h urine volume output and by increasing albumin/creatinine ratio in *ob/ob* in comparison to paired C57BL/6 mice; 2) renal dysfunction occurred in parallel to exaggerated effect of VitD_3_ in increasing intrarenal VC in *ob/ob* mice *vs*. C57BL/6 mice; 3) Increased BMP-2 expression in the vascular wall of calcified intra-renal arteries from *ob/ob* mice, but not from C57BL/6 animals after VitD_3_ protocol; 4) increased mesangial expansion in *ob/ob* mice, without an additive effect of VitD_3_, associated with severe acute tubular necrosis in VitD_3_-treated *ob/ob* mice, contributing to higher levels of albuminuria, and decreased kidney function.

Hypercalcemia, due to increased intestinal and renal calcium resorption is an expected effect of VitD_3_^25,26^ supplementation at high serum levels^27^. Hypercalcemia has been extensively studied in this context, since this is a mechanism involved in VC due to high calcium x phosphorus product^14,28^. To further understand increased VC response of *ob/ob* mice after our protocol, we performed serum biochemical analysis and assessed bone histomorphometric parameters. We found a significant, but similar increase in serum calcium concentration (≈60% increase), both in *ob/ob* and in C57BL/6 mice after VitD_3_ administration when compared to saline-injected mice^14^. Moreover, both VitD_3_ and kidney dysfunction-induced effect on bone tissue damage, observed in our study, are demonstrated by increased osteoid matrix and resorption parameters, which characterizes a mixed bone disease^29^. Bone disease, as shown by parameters of bone resorption (e.g. osteoblastic surface, resorption surface, and diminished number of osteoclasts) was similar in both strains. This implies the reproducibility of the effect of VitD_3_ in bone/calcium metabolism both in *ob/ob* and in C57BL/6 mice, conceiving the idea that augmented VC in *ob/ob* mice translates an individual response from the obese insulin resistance model. A limitation of our study is that we were not able to precisely determine absolute values of serum 25-hidroxyvitamin D both in C57BL/6 and in *ob/ob* mice after VitD_3_ stimulation. In this setting, we found that 25-hidroxyvitamin D serum concentration increased to above 100 ng/mL (the limit of the standard curve used, data not shown) in both strains after excessive VitD_3_ supplementation. In fact, although we used less VitD_3_ in *ob/ob* mice *versus* C57BL/6 mice, these animals showed increased calcification of intrarenal arteries associated with greater kidney dysfunction in comparison to C57BL/6 mice.

VC, which is a condition without specific medical treatment, positively associates with coronary artery disease and cardiovascular events, especially in patients with diabetes mellitus^30^. Interestingly, now we showed an association of augmented intra-renal arteries calcification and renal dysfunction in *ob/ob* mice, demonstrated by positive correlation between intrarenal arteries calcification and increased albumin/creatinine ratio, and decreased creatinine clearance. This experiment suggests a direct relationship between arterial calcification and decreased renal function induced by hypervitaminosis D. Furthermore, we demonstrated that high-dose VitD_3_ stimulation promoted a paradoxical increase in mesangial area in wild type mice. Thus, our work unveils an important effect of VitD_3_ on mesangial compartment in a toxic dose-dependent manner. We postulate that this effect may include VDR aberrant down-regulation in that compartment and probably in podocytes as well. In *ob/ob* mice, VitD_3_ treatment was not implicated in an additive effect in mesangial compartment expansion and in glomerular area augmentation, which may be explained at least by the fact that animals were not treated with insulin and hyperglycemia may continuously aggravated mesangial expansion and abrogated VitD_3_-mediated renoprotective effects. Furthermore, VitD_3_ toxicity may induce renal hemodynamic dysfunction, which can be complicated by ATN. Tubular damage could also be explained by diabetes-induced lysosomal dysfunction in proximal tubules^31^. Therefore, as a surrogate marker of diabetic kidney disease progression, the augmentation in exocytosis-mediated urinary megalin excretion creates a vicious cycle of tubular damage and may also contribute to higher values of albuminuria found in VitD_3_-treated *ob/ob* mice. Other possible mechanisms include acute hypercalcemia induced by VitD_3_ toxicity, which may lead to acute kidney injury by decreasing extracellular fluid volume due to anorexia, nausea, vomiting, and decreased ability to concentrate urine, besides a direct renal vasoconstriction effect, as observed in VitD_3_-treated animals from our protocol, and in vitamin D intoxication in humans^13^. VitD_3_ effects are mediated by VDR. Previously, we demonstrated that VDR downregulation was abrogated in *ob/ob* mice after VitD_3_ stimulation, which corroborated to increased VC (aorta) in this mouse model^14,15^. In the kidneys, VDR is mainly expressed in proximal and distal tubular epithelial cells, podocytes, macula densa of the juxtaglomerular apparatus, and collecting duct epithelial cells^32^. However, VDR expression is low in glomerular mesangial cells. Despite the low expression of VDR in mesangial compartment, VitD_3_ may reduce mesangial cell proliferation induced by hyperglycemia in diabetic rat via mTOR pathway modulation and decreasing glomerular volume^33^. In addition, VitD_3_ suppressed Monocyte Chemoattractant Protein-1 (MCP-1) expression in mesangial cells by blocking Nuclear factor kappa B (NF-κB) activation, which indicates that vitamin D may protect the kidney by reducing macrophage infiltration^34^. Moreover, VitD_3_ *per se* was able to increase VDR expression at mRNA and protein levels in mesangial cells cultured with either low or high glucose^34^.

Diabetes mellitus may activate specific osteochondrogenic signaling involved in vascular smooth muscle cells dedifferentiation into osteoblast-like cells, thus increasing VC progression. To further investigate this specifically in intrarenal arteries, we assessed BMP-2 expression in the kidney. BMP-2 is a protein secreted by smooth muscle vascular cells, endothelial cells and inflammatory macrophages^35^. BMP-2 binds to its receptor on the plasma membrane, triggers a specific intracellular signaling cascade, activating osteochondrogenesis and VC. Of note, BMP-2 expression is also regulated by the BMP signaling pathway itself, since BMP-2 is a self-regulatory protein^36^. In diabetic preclinical animal models, VC is governed by increased MSH homeobox 2 (Msx2) and Tumor Necrosis Factor Alpha (TNF-alpha) expression in the middle layer and in the adventitia of vessel wall, finally activating osteochondrogenic transcription program in smooth muscle cells. Previous reports demonstrated that increased macrophage infiltration in the adipose tissue and in the kidney, as well as augmented TNF-alpha expression in animal models of obesity, play a pivotal role in inducing kidney disease^37,38^. Accordingly, inflammatory cytokines e.g. C-C chemokine ligand 2 (CCL2) initiates inflammation by binding to C-C chemokine receptor 2 (CCR2) to induce diabetic glomerular sclerosis^39^. Moreover, investigators showed that blocking CCL2/CCR2 signaling pathway can ameliorate renal injury and proteinuria in a mouse model of obesity and insulin resistance^37^. BMP-2 activation has also been well characterized in aorta from diabetic mice^40^. In our laboratory, vascular smooth muscle cells isolated from *ob/ob* mice aortae showed increased calcification *in vitro* after BMP-2 stimulation with concurrent upregulation of other osteogenic proteins^41^. Accordingly, we showed augmented baseline MSX2, BMP-2 expression and Smad 1,5 phosphorylation as well as increased BMP-2-induced osteocondrogenic signaling and dedifferentiation in *ob/ob* vascular smooth muscle cells in comparison to paired C57BL/6 vascular smooth muscle cells^41^. In the present study, we identified a significant increase in BMP-2 expression in calcified intrarenal arteries of VitD_3_-treated *ob/ob* mice, which also demonstrated renal dysfunction. On the contrary, paired VitD_3_-treated C57BL/6 mice demonstrated that BMP-2 expression was very low in intrarenal arteries. We speculate that VitD_3_ induces BMP-2 expression, through the activation of its receptor. To assess the role of leptin-deficiency in vascular smooth muscle cells calcification from *ob/ob* mice, we demonstrated that calcification increased in leptin-incubated *ob/ob* vascular smooth muscle cells, but not in leptin-incubated C57BL/6 vascular smooth muscle cells (Supplemental Fig. II). On the contrary of C57BL/6 vascular smooth muscle cells, co-incubation of leptin in BMP-2-treated cells further augmented mineralization in *ob/ob* vascular smooth muscle cells. These data suggest mechanisms of increased susceptibility of *ob/ob* mice to develop accelerated vascular calcification, independently of leptin supplementation. A putative explanation for the exaggerated response of *ob/ob* mice could be that CYP27B1 (1-α-hydroxylase) and CYP24A1 (24-hydroxylase), which are enzymes responsible for regulating active VitD_3_ metabolites, exhibit altered mRNA and activity levels, thus favoring increased local concentration of bioactive VitD_3_ and consequently potentiating BMP-2 expression in leptin-deficient diabetic mice. Of note, non-hepatic 25-hydroxylases (Cyp2R1 and Cyp27A1), enzymes responsible for the conversion of cholecalciferol into 25-hydroxyvitamin D and for maintaining VDR levels^42^, may have contributed to changes in VDR expression levels and the phenotype demonstrated in our model, because 25-hydroxylases lack the tight control that exists for 1-hydroxylase and 24-hydroxylase during excessive cholecalciferol supplementation. Altogether, we postulate that augmented intra-renal calcification potentiates progression of renal dysfunction in *ob/ob* mice. Moreover, a mechanism involved could be worsening of Windkessel effect, because of increased arterial rigidity and decreased vascular elasticity, which impairs tissue perfusion due to VC^24,43^. In addition, vascular mineralization may act as an adjunct etiology of this process or even in its progression^44,45^, adding importance to this study. Nonetheless, we did not find glomerular calcification in *ob/ob* mice, but we cannot rule out the hypothesis that decreased tissue perfusion and increased BMP-2 expression could have impacted in glomerular dysfunction, by altering podocytes number and/or function^46–48^ and increasing inflammation^19,49^. A limitation of our study is that we can’t distinguish whether systemic and local VitD_3_ effects, e.g. increased serum calcium levels, augmented BMP-2 expression and intrarenal arteries calcification influenced renal dysfunction alone or whether this occurred together with hemodynamic imbalance due to vascular mineralization. This needs further clarification by exploring respective individual impact on renal disease progression. In conclusion our results demonstrate that high-dose VitD_3_ administration in *ob/ob* mice, but not in C57BL/6, induce increased intrarenal VC associated with kidney dysfunction. These conditions are common in diabetic patients, bringing high morbidity and mortality^50,51^. Moreover, pathophysiological and molecular mechanisms investigated in this study represent an important contribution both to understand VitD_3_ biological properties in the kidneys and to clarify specific aspects of renal disease in diabetic patients, which usually present with VC^7,8^. Our model may be instrumental for the investigation of new therapeutic targets and/or to develop compounds that attenuate renal dysfunction in diabetes mellitus, especially in the context of increased VC.

## Materials and Methods

### Animals

We used 16 to 20-week-old male homozygous leptin-deficient *ob/ob* mice (C57BL/6 background) from Jackson Laboratory (Bar Harbor, ME). A total of 30 *ob/ob and 30* C57BL/6 littermates were used to perform the experiments. This study was conducted after approval of the protocol #2242-14 by Sociedade Beneficiente Israelita Albert Einstein’s ethics committee and undertook according to guidelines for the care and use of laboratory animals, which conforms to Guide for the Care and Use of Laboratory Animals (NIH Publication, 8^th^ edition). After 21 days, animals were euthanized with 1 mg/kg IM xylazine chlorohydrate (Bayer, São Paulo, Brazil, Cat#1002181) and 100 mg/kg IM ketamine chlorohydrate (Cristália, São Paulo, Brazil, Cat#404800). Of note, not all 60 animals were represented in all experiments, due to technical difficulties as follow: (i) we were not able to draw blood samples from all animals (dehydration, samples with clots, unsuccessful vein puncture); (ii) automated Abbott^®^ i-STAT Clinical Analyzer failed to give a result in some samples (equipment error) and repeated measurement were not possible due to insufficient sample/blood volume; (iii) we did not have as many metabolic cages as the number of animals used during protocol, in order to collect 24h-urine from all animals; (iv) some animals were anuric, so we were not able to determine albuminuria in some samples/animals, (v) we did not use all animals to perform all the experiments.

### Vitamin D_3_ administration protocol

*Ob/ob* and C57BL/6 male mice were injected with VitD_3_ 6.4 × 10^4^ IU/day and 4.4 × 10^4^ IU/day respectively i.p. for 18 days and another 3 days of sodium chloride 0.9% i.p. This corresponds to a daily dose of 1.46 × 10^3^ IU/g/day administered to C57BL/6 mice and 1.06 × 10^3^IU/g/day administered to *ob/ob* mice, considering a body weight of 30 g and 60 g respectively. Sodium chloride 0.9% (saline) i.p. only was used for 21 days in controls. Of note, we did not use the same proportional dose (calculated by body weight) in *ob/ob* and in C57BL/6 mice, because 80% of *ob/ob* mice died when we used the aforementioned proportional dose, due to Vitamin D intoxication after 7 to 10 days of protocol. Consequently, we reduced the dose in approximately 30% in *ob/ob* mice to be able to complete the animal protocol.

### Serum and urine analysis

Blood was collected from the animals before and after the protocol in order to assess serum glucose, urea, creatinine and calcium levels using Abbot^®^ i-STAT Clinical Analyzer and the I-STAT CHEM8+ cartridge (Abbott Laboratories, Illinois, USA, Cat#AB-9P3125). For urine analysis, animals were maintained in a metabolic cage to collect 24-hour urine before and after the protocol. Urine albumin levels were determined by Albumin Mouse ELISA Kit (Abcam, Cat#ab108792). Urine creatinine levels were evaluated by a colorimetric assay Labtest Diagnostics kit (Vista Alegre, Brazil, Cat#10009010034) and quantified by an automatic biochemical analyzer Cobas Mira Plus (Roche, Switzerland).

### Bone histomorphometric analysis

After euthanasia, left femurs were dissected, fixed in 70% ethanol, dehydrated, embedded in methyl methacrylate, and sectioned longitudinally with a Policut S microtome (Reichert-Jung, Heidelberg, Germany) in 5 µm-thick sections. Samples were stained with 0.1% toluidine blue (pH 6.4) for histomorphometric analysis which was performed using semiautomatic method with a Labophot-2A microscope (Nikon®), and software Osteomeasure (Osteometrics, Inc, Atlanta, EUA). These histomorphometric parameters are suggested by the American Society of Bone and Mineral Research histomorphometry nomenclature committee^52^ as follow: bone volume represented as a percentage of tissue volume (BV/TV, %); trabecular thickness (Tb.Th, µm); trabecular separation (Tb.Sp, µm); trabecular number (Tb.N, /mm); osteoid volume as a percentage of bone volume (OV/BV, %); osteoid surface as a percentage of bone surface (OS/BS, %); osteoblast surface as a percentage of bone surface (Ob.S/BS, %); osteoid thickness (O.Th, µm); osteoclast surface as a percentage of bone surface (Oc.S/BS, %); and eroded surface as a percentage of bone surface (ES/BS, %).

### *Ex vivo* calcification assessment of the kidneys using Osteosense 680 EX

24 h before sacrifice, mice were injected with 0.2 µM Osteosense 680 EX (NEV10020EX, Perkin Elmer, USA). After euthanasia, cardiovascular system from mice was perfused with saline, followed by radical nephrectomy. Isolated kidneys were analyzed with a fluorescence detector IVIS ® Lumina LT Series III (PerkinElmer, USA), and fluorescence signals were normalized to estereoradian and ROI of 4.0 cm^2^ and converted to photon/s^53^.

### Histological quantification of kidneys’ vascular calcification

Kidneys were fixed in 10% phosphate-buffered formalin (pH 7.4), embedded in paraffin and processed for Von Kossa (silver nitrate Sigma, Cat# S1179) and Alizarin Red S (Sigma, Cat#A5533) analysis using a FSX100 microscope (Olympus Life Sciences) and Olympus CellSens software. Kidneys from animals previously labeled with Osteosense 680 EX, were extracted, incubated with sucrose (Sigma, Cat#S9378) 30% overnight, frozen at −80 °C in O.C.T. (optimum cutting temperature) compound (Sakura Finetek, California, USA, VWR Cat# 25608-930) and sections were obtained using a cryostat microtome (Leica Biosystems, Germany). Samples were incubated with Hoechst 33342 (Thermo Fischer Scientific, Cat#H1399) for nuclei staining, and analyzed by Zeiss LSM 710 laser scanning microscope for confocal imaging and the respective software Zen Image (Zeiss®). Osteosense fluorescence quantification was calculated by using the fluorescence intensity divided by vascular area and normalized by C57BL/6 control.

### Immunofluorescence analysis of Bone Morphogenetic Protein-2 (BMP-2) expression in the vascular wall

10 µm O.C.T. kidney’s sections were fixed with 4% paraformaldehyde and permeabilized with 0.25% Triton X-100 and PBS for 15 min. Samples were washed, incubated with primary antibody overnight (anti-BMP-2, Abcam, ab:6285) 5 µg/mL, and finally incubated with secondary anti-mouse IgG AlexaFluor 488 (Thermo Fischer Scientific, Cat#A10680) 10 µg/mL for 1 hour, and coverslipped with Hoechst 33342 (Thermo Fischer Scientific, Cat#H1399). Fluorescence image analysis was performed in parallel with controls, using the same settings for all samples in a Zeiss LSM 710 confocal laser scanning microscope and the respective software Zen Image (Zeiss®). BMP-2-derived fluorescence quantification was calculated by using the fluorescence intensity divided by vascular area.

### Mesangial and histological assessment of the kidneys

Kidney sections were stained with periodic acid-Schiff (PAS) trichrome staining in each experimental group. Sections were then analyzed by light microscopy (magnification, x400). A quantitative analysis of mesangial expansion was performed. The increase in mesangial matrix was determined by the presence of PAS-positive area in the mesangium, and was expressed in percentage. The glomerular area (μm^2^) was also traced along the outline of capillary loops using CellSens software (Olympus) in 28–35 randomly selected glomeruli in each animal. Acute tubular necrosis (ATN) was identified by PAS staining, followed by quantification of the following variables: presence of casts, brush border loss, tubular dilation, necrosis, and calcification. Picro Sirius Red staining was assessed by standard light microscopy (magnification, x100).

### Statistical analysis

Data are shown as mean ± standard error of the mean (M ± S.E.M). After assessing normality and equal variance, data were analyzed by One-Way ANOVA followed by Tukey test to compare three or more groups, or paired T-test to compare two groups, considering statistically significant if *P* < 0.05. We used GraphPad Prism 5.0 software (GraphPad Software Inc., La Jolla, CA, USA).

## Supplementary information

Supplementary  Information

## Data Availability

The datasets generated during and/or analysed during the current study are available in 1.Liberman, M. Dataset Almeida *et al*.xlsx. (2019). 10.6084/m9.figshare.7949453.v5.
