# Supplementary material for: Excessive cholecalciferol supplementation increases kidney dysfunction associated with intrarenal artery calcification in obese insulin-resistant mice
Source: Sci Rep. 2020 Jan 9;10:87. doi: 10.1038/s41598-019-55501-3 (PMC6952360; doi:10.1038/s41598-019-55501-3)
Supplement: Supplementary file 1 — Supplementary Information [file 41598_2019_55501_MOESM1_ESM.docx]

# Excessive cholecalciferol supplementation increases kidney dysfunction associated with intrarenal artery calcification in obese insulin-resistant mice

**Youri E. Almeida, Melissa R. Fessel, Luciana Simão do Carmo, Vanda Jorgetti, Elisângela Farias-Silva, Luciana Alves Pescatore, Lionel F. Gamarra, Maria Claudina Andrade, Antonio Simplicio-Filho, Cristóvão Luis Pitangueiras Mangueira, Érika B. Rangel and Marcel Liberman**

**Supplemental Material**

**Expanded Methods**

**Vascular smooth muscle cells culture**

Vascular smooth muscle cells (VSMC) were isolated either from 15-week-old male homozygous leptin-deficient *ob/ob* mice aorta C57BL/6 background (The Jackson Laboratory, Bar Harbor, ME) or from paired C57BL/6 control littermates aorta as previously described.^1^ Passages 4 to 8 were used for the experiments. C57BL/6 and *ob/ob* VSMC were cultured in DMEM + FBS 5%, which was replaced each 2 days and incubated without or with BMP-2 50ng/mL to investigate VSMC calcification after 14 days. Leptin 10ng/mL was added in specific experiments. No additional calcifying medium was supplemented.

**Assessment of vascular smooth muscle cells calcification**

VSMC calcification was detected by Alizarin Red S pH 4.1 and quantified by spectrophotometry (550 nm) after extracting the staining with CPC 10% (cetylpyridinium chloride) in sodium phosphate pH 7, normalized by protein content.^2^


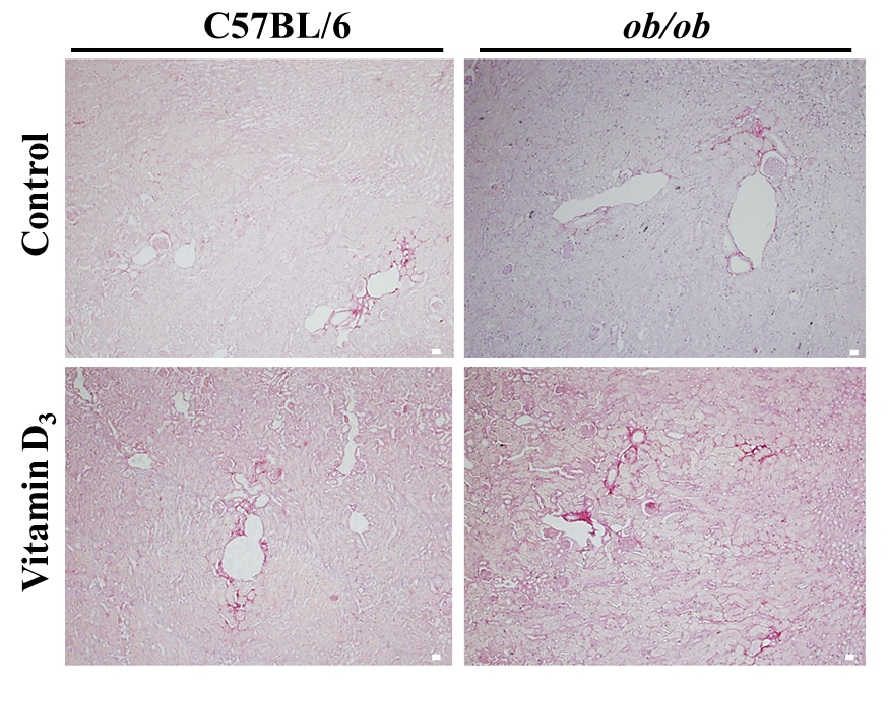


**Supplemental Figure I– Picro Sirius Red staining demonstrated augmented collagen I and collagen III deposition in tubule-interstitial area after vitamin D_3_ protocol.** Picro Sirius Red staining of C57BL/6 and *ob/ob* mice after VitD3 protocol or saline (control). Increased Picro Sirius staining in VitD_3_-treated *ob/ob* mice. Bars=20 µm. (saline *n*=4-6; VitD_3_ *n*=5-8).


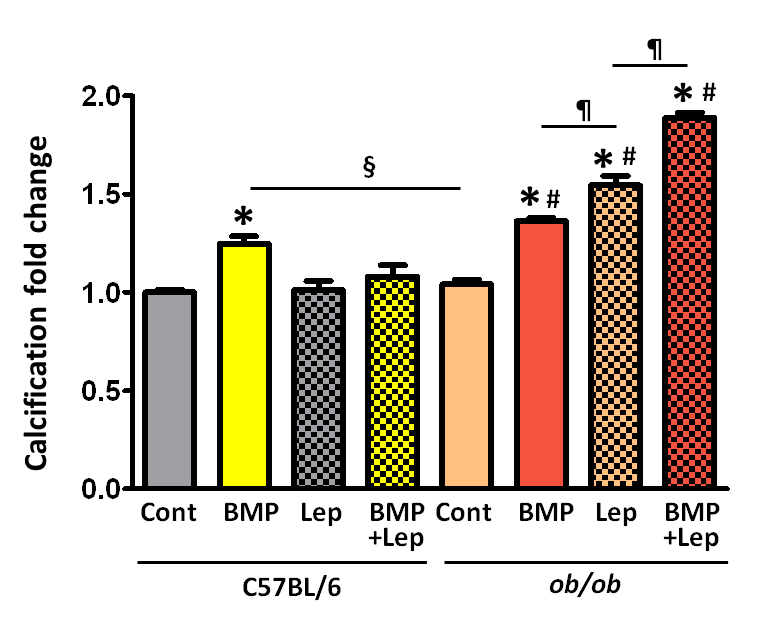


**Supplemental Figure II- Increased calcification in *ob/ob* vascular smooth muscle cells (isolated from aorta).** *Ob/ob* vascular smooth muscle cells calcification augmented either after BMP-2 50ng/mL or after leptin 10ng/mL incubation for 14 days in comparison to paired C57BL/6 vascular smooth muscle cells. Mineralization was additionally increased when leptin was co-incubated with BMP-2 in *ob/ob* vascular smooth muscle cells, but not in C57BL/6 vascular smooth muscle cells. **P*<0.05 vs. C57BL/6 Control; ¶*P*<0.05 ANOVA; #*P*<0.05 vs. C57BL/6 (all groups) ANOVA and vs. *ob/ob* Cont; §*P*<0.05 ANOVA; n=3-6.

**References**

1. Andrade MC, Carmo LS, Farias-Silva E, Liberman M. Msx2 is required for vascular smooth muscle cells osteoblastic differentiation but not calcification in insulin-resistant ob/ob mice. *Atherosclerosis.* 2017;265:14-21.

2. Stanford CM, Jacobson PA, Eanes ED, Lembke LA, Midura RJ. Rapidly forming apatitic mineral in an osteoblastic cell line (UMR 106-01 BSP). *The Journal of biological chemistry.* 1995;270(16):9420-9428.
